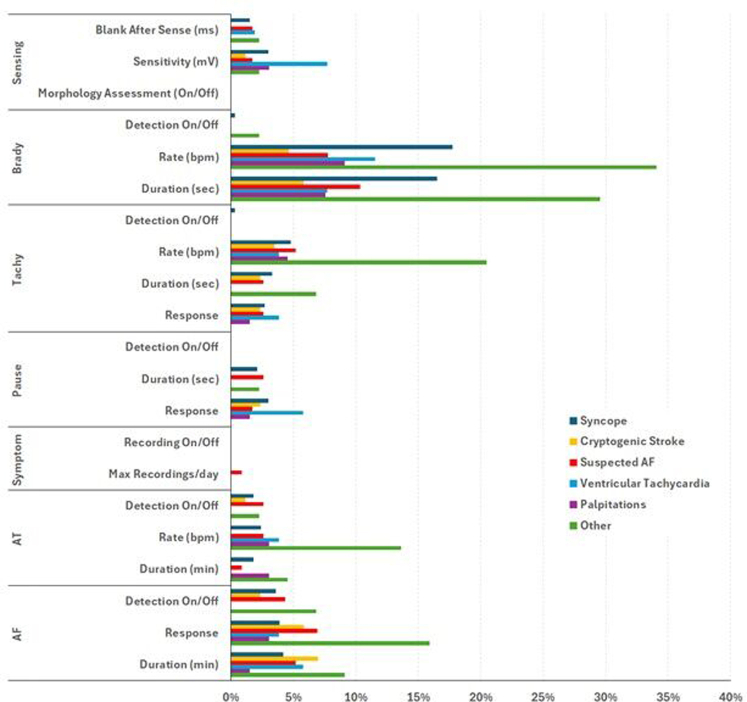# Real-world use of insertable cardiac monitor remote programming: A multicenter European experience

**DOI:** 10.1016/j.hroo.2025.08.035

**Published:** 2025-09-01

**Authors:** Samir Fareh, Stefano Nardi, Luigi Argenziano, Luca Poggio, Alessandro Costa, Fernando Scala, Alessandro Diamante, Giovanni Luzzi, Carlo Lavalle, Luca Checchi, Michele Magnocavallo, Massimo Stefano Silvetti, Daniele Porcelli, Domenico Gianfrancesco, Andrea Boncompagni, Paul Charles, Vittoria Marino, Monica Campari, Sergio Valsecchi, Giulio Conte

**Affiliations:** 1 Hôpital de la Croix-Rousse et Hôpital Lyon Sud, Hospices Civils de Lyon, Lyon, France; 2 Pineta Grande” Hospital, Castel Volturno, CE, Italy; 3 Clinica Sanatrix, Naples, Italy; 4 Ospedale Maggiore Di Lodi, Lodi, Italy; 5 Ospedale Sacro Cuore - Don Calabria, Negrar (VR), Italy; 6 Fatebenefratelli Hospital, Naples, Italy; 7 Casa Di Cura “Villa Azzurra,”Siracusa, Italy; 8 Santissima Annunziata” Hospital, Taranto, Italy; 9 “Sapienza” University of Rome, Policlinico Umberto I, Rome, Italy; 10 Arrhythmia and Electrophysiology Unit, Careggi University Hospital, Florence, Italy; 11 Ospedale Isola Tiberina – Gemelli Isola, Rome, Italy; 12 Bambin Gesu” Pediatric Hospital, Rome, Italy; 13 San Pietro-Fatebenefratelli Hospital, Rome, Italy; 14 “L. Bonomo” Hospital, Andria, Italy; 15 Ospedale S. Jacopo, Pistoia, Italy; 16 Boston Scientific Italia, Milan, Italy; 17 Cardiocentro Ticino Institute, Ente Ospedaliero Cantonale, Lugano, Switzerland; 18 Faculty of Biomedical Sciences, Università della Svizzera Italiana, Lugano, Switzerland

**Keywords:** Insertable cardiac monitor, Loop recorder, Arrhythmias, Syncope, Atrial fibrillation, Cryptogenic stroke

## Abstract

**Background:**

Insertable cardiac monitors (ICMs) enable continuous arrhythmia monitoring but generate high transmission volumes, increasing clinical workload. The LUX-Dx ICM (Boston Scientific) allows remote reprogramming of device alert settings, potentially reducing in-office visits.

**Objective:**

This study aimed to evaluate the real-world use of remote reprogramming after its initial commercialization in Europe and its impact on transmission burden.

**Methods:**

Deidentified data were collected from 697 consecutive patients across 23 European centers between 2022 and 2024.

**Results:**

Syncope (48%) was the most frequent indication for ICM implantation. Patients were followed for a median of 9 months (25th–75th percentile 4–13). A total of 401 reprogramming events (0.8 per patient-year) occurred in 230 ICMs, with 38% (95% confidence interval 34–43) of devices reprogrammed within 1 year. Of these, 156 (39%) were performed remotely. The overall transmission rate was 3.0 per patient-month (95% confidence interval 2.9–3.1): alert transmissions (64%), scheduled transmissions (31%), patient-initiated interrogations (4%), and clinician-initiated interrogations (1%). The rate of recorded episodes varied significantly by indication (*P* < .001), with bradycardia the most frequent across groups. Reprogramming significantly reduced transmission rates (median 57%; 25th–75th percentile 4–86), alerts (78%; 25th–75th percentile 11–96), and recorded episodes (91%; 25th–75th percentile 47–99) (all *P* < .001).

**Conclusion:**

ICM reprogramming plays a key role in optimizing device performance and reducing remote monitoring burden. Currently used in 39% of cases, remote reprogramming holds potential for broader adoption to minimize in-office visits. Efficiency may be further improved by transitioning to an alert-based monitoring strategy and eliminating scheduled transmissions.

## Graphical abstract

**Figure:**
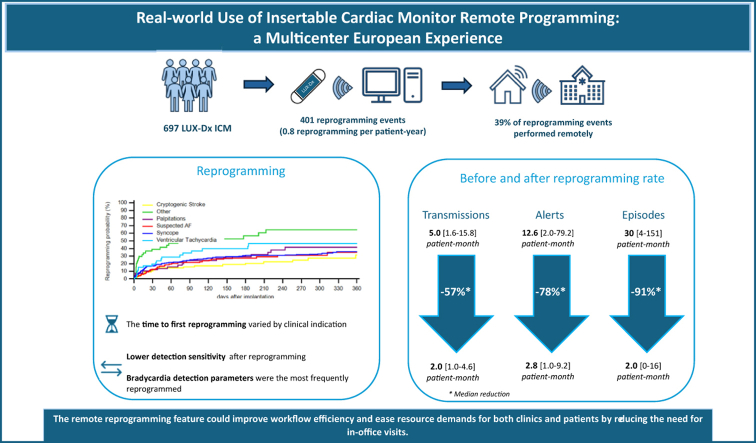

**Key Findings**

▪ Insertable cardiac monitor (ICM) reprogramming optimizes detection settings and significantly reduces workload by lowering unnecessary transmissions.
▪ ICM remote reprogramming minimizes in-office visits, reducing staff time and hospital workload and optimizing resource allocation.
▪ Alert-based monitoring strategies can reduce the follow-up workload by up to one-third by eliminating scheduled transmissions.

## Introduction

The use of insertable cardiac monitors (ICMs) for long-term continuous monitoring of cardiac arrhythmias has increased significantly in recent years. However, each ICM transmission must be manually reviewed by clinical staff to confirm accuracy, leading to a substantial workload in remote monitoring clinics.^1^^,^^2^ The high volume of transmissions is driven by both automated alerts and patient-activated events, with transmission frequency largely influenced by programming settings, the indication for monitoring, and patient education.

To optimize the diagnostic value of ICMs, appropriate alert programming is essential, as emphasized in the recently published Expert Consensus Statement on the Practical Management of Remote Device Clinics.^2^ Reprogramming ICMs after identifying false-positive or nonactionable transmissions can reduce unnecessary alerts and improve efficiency. In addition, reprogramming after a true-positive transmission may allow for the monitoring of arrhythmia progression after an initial diagnosis has been established. Recent advancements, such as remote programming capabilities, now allow clinicians to make iterative adjustments to ICM settings without requiring in-person visits.^3^^,^^4^

The LUX-Dx (Boston Scientific) is a novel ICM that incorporates dual-stage arrhythmia detection algorithms and remote programming functionality.^5^^,^^6^ This study aimed to characterize the real-world use of remote reprogramming during its initial commercialization in Europe and evaluate its impact on ICM transmission burden.

## Methods

### Study design

From October 2022 to December 2024, consecutive patients undergoing implantation of a LUX-Dx ICM were retrospectively included across 23 European centers (see Appendix). The decision to implant the device was at the discretion of the operator, with no standardized indication agreed upon among the participating centers. Devices were implanted and programmed according to each center’s routine clinical practice. Similarly, postimplantation follow-up was conducted in accordance with standard practice at each site. The follow-up period began on the day of ICM implantation and continued until the most recent data transmission.

The primary objective of this study was to characterize the use of the remote programming feature during the initial commercialization of the LUX-Dx ICM in Europe and assess its impact on transmission burden. To achieve this, we analyzed the overall use of remote reprogramming, including the time to first reprogramming, and quantified the proportion of reprogramming events completed remotely. In addition, we categorized the parameter changes performed both in-clinic and remotely. Reprogramming changes were classified based on their impact on detection sensitivity, that is, alert criteria made less or more stringent. Furthermore, we measured the total number and frequency of remote transmissions, including both alert-triggered and scheduled transmissions, throughout the monitoring period. Transmission rates were compared before and after reprogramming to assess the impact of parameter adjustments.

The estimations of the staff time required to manage ICMs and the potential time savings with remote reprogramming and programming optimization were based on a previous analysis that quantified the average staff time required to review remote transmissions (11.3 minutes) and manage in-person clinic visits (39.9 minutes) in Europe.^1^ For the analysis, we assumed 2000 hours annually per full-time equivalent.

This was a retrospective analysis of aggregated data from the Boston Scientific LATITUDE remote monitoring system collected in real-world clinical practice. The research reported in this paper adhered to the Declaration of Helsinki. The study was exempt from institutional review board approval at participating institutions. Data processing was conducted in compliance with the European General Data Protection Regulation (EU 2016/679), ensuring full anonymization of personal health data as mandated by European regulations. All patients had provided a written consent for data use at the time of remote monitoring activation. This study was not industry funded.

### The device

The LUX-Dx is a small ICM designed to monitor, record, and store data related to cardiac arrhythmias that fall into 5 categories: pauses, bradyarrhythmias, tachyarrhythmias, atrial fibrillation (AF), and atrial tachycardia. Each category’s algorithm contains settings that can be tailored according to the patient’s specific clinical indications. The LUX-Dx ICM provides remote programming capabilities. After the ICM insertion, patients receive a mobile device with the preloaded myLUX Patient App, designed to activate the patient’s implanted ICM and transmit data between their ICM and the LATITUDE server. The application also offers other user-friendly features (notifications, instructions, and educational material) intended to empower patients and increase compliance.

### Statistical analysis

Quantitative variables are reported as means ± standard deviations if normally distributed or medians with 25th to 75th percentiles in the case of skewed distribution. Normality of distribution was tested using the nonparametric Kolmogorov–Smirnov test. Categorical data were expressed as percentages. Event rates were calculated as the ratio between the total count of events and the respective patient follow-up durations and were expressed as events per patient-month or patient-year. Analysis of the time to the first event was made using the Kaplan–Meier method, and the log-rank test was applied to evaluate differences between trends. *P* < .05 was considered significant for all tests. All statistical analyses were performed using R: a language and environment for statistical computing (R Foundation for Statistical Computing).

## Results

### Study population and device programming

A total of 697 consecutive implantation procedures were successfully conducted across 23 European centers. Syncope (48%) was the most frequent indication for ICM implantation, followed by suspected AF (17%) (Table 1). The programming of ICM settings at implantation is presented in Supplemental Table 1. Scheduled device transmissions were activated in all patients and usually programmed at least once every 30 days.

**Table 1:** Baseline clinical parameters and indications for ICM implantation

Parameter	n = 697
Men, n (%)	391 (56)
Age, y	64 ± 18
Reason for monitoring, n (%)	
Syncope	333 (48)
Cryptogenic stroke	86 (12)
Suspected atrial fibrillation	116 (17)
Ventricular tachycardia	52 (7)
Palpitation	66 (9)
Other	44 (6)

ICM **=** insertable cardiac monitor.

### Follow-up

**Figure 1:**
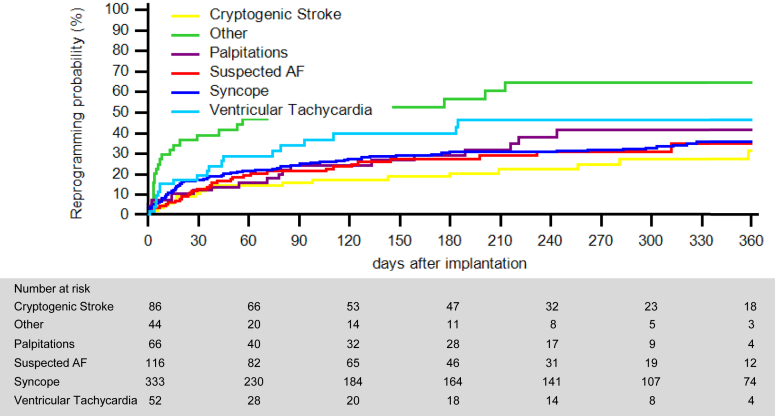
Time to first reprogramming. Patients are stratified according to the indications for insertable cardiac monitor implantation. AF = atrial fibrillation.

**Figure 2:**
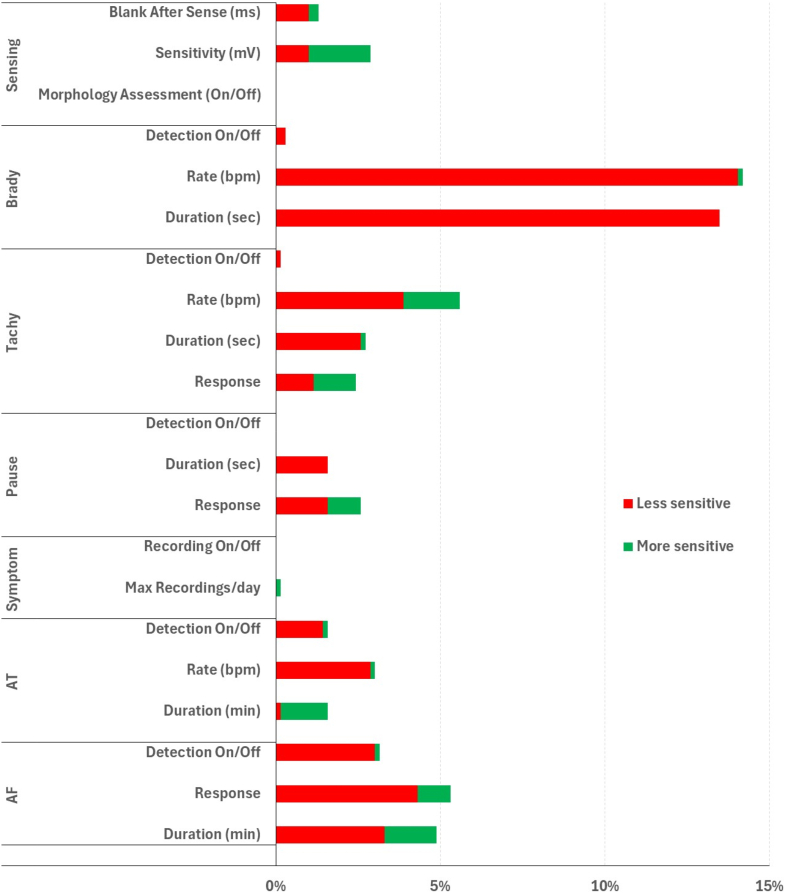
Percentage of patients with parameter settings changed from implantation programming to the last transmission. In red are reported changes resulting in lower sensitivity (detection and alert criteria made more stringent); in green are changes resulting in higher sensitivity (criteria less stringent). AF = atrial fibrillation; AT = atrial tachycardia.

Patients were followed for a median of 9 months (25th–75th percentile: 4–13). A total of 401 reprogramming events (0.8 per patient-year) were performed in 230 patients, with 156 events (39%) conducted remotely and 245 in-office. Seventeen percent of ICMs (95% confidence interval [CI] 14–20) were reprogrammed within 30 days after insertion, and 38% (95% CI 34-43) within 1 year. The time to first reprogramming varied by clinical indication (*P* < .001) (Figure 1). The details of the parameter settings changed from implantation programming to the last transmission are presented in Figure 2. The vast majority of changes were in the direction of a lower detection sensitivity, that is, more stringent alert criteria, and most frequently involved the parameters for bradycardia detection. The analysis stratified according to the indications for ICM implantation revealed a higher frequency of changes in patients implanted for syncope or reasons classified as “other” (Supplemental Figure 1).

**Figure 3:**
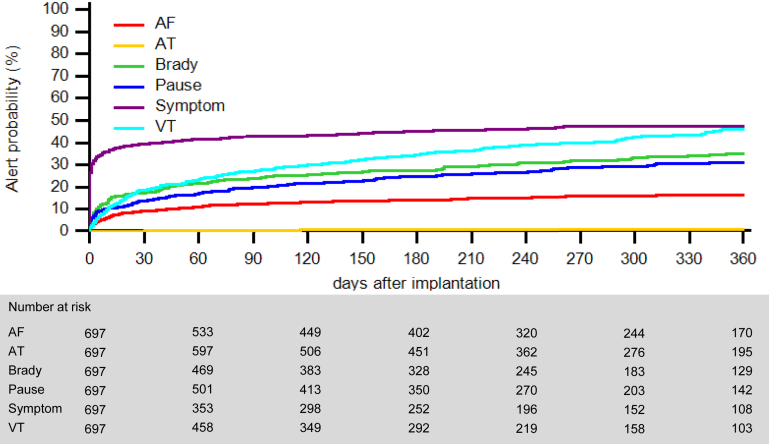
Time to first alert transmission for different reasons. AF = atrial fibrillation; AT = atrial tachycardia; VT = ventricular tachycardia.

**Figure 4:**
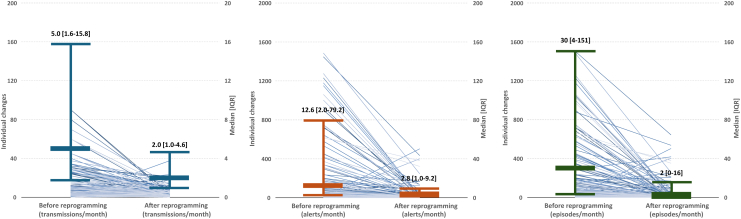
Rates of transmissions, alerts, and recorded episodes before and after reprogramming for patients who underwent reprogramming.

Over the follow-up period, 18,668 device transmissions were received, that is, 3.0 per patient-month (95% CI 2.9–3.1). Transmission rates varied by indication, from 2.1 per patient-month for ventricular tachycardia (VT) to 4.3 per patient-month for palpitations (*P* < .001) (Table 2). Alert transmissions accounted for 64% (11,944), scheduled transmissions for 31% (5832), patient-initiated interrogations for 4% (727), and clinician-initiated interrogations for 1% (165). The time to the first alert for different reasons is shown in Figure 3. The rates of recorded episodes also varied significantly by indication (*P* < .001), but bradycardia was the most frequent detection condition for all indication groups (Table 2). In patients who underwent reprogramming, a paired analysis showed significant reductions of the rate of transmissions (median 57%; 25th–75th percentile 4–86), alerts (median 78%; 25th–75th percentile 11–96), and recorded episodes (median 91%; 25th–75th percentile 47–99), after reprogramming (all *P* < .001) (Figure 4).

**Table 2:** Events occurred during follow-up

	Transmissions/patient-mo	Alerts per transmission	Episodes/patient-mo (95% CI)						
	(95% CI)	Median [IQR]	AF	AT	Brady	Pause	Symptom	VT	Total
Cryptogenic stroke	2.4 (2.3–2.6)	1 [1–3]	4.4 (4.3–4.6)	0.1 (0.1–0.1)	16.8 (16.5–17.1)	2.0 (1.9–2.1)	0.5 (0.4–0.5)	1.5 (1.4–1.6)	25.3 (25.0–25.7)
Other	2.9 (2.7–3.1)	1 [1–1]	10.0 (9.7–10.3)	0.2 (0.2–0.3)	47.5 (46.8–48.2)	0.9 (0.8–1.0)	0.5 (0.4–0.5)	1.2 (1.1–1.3)	60.2 (59.4–61.0)
Palpitations	4.3 (4.1–4.5)	1 [1–3]	1.4 (1.3–1.5)	0.1 (0.1–0.1)	13.8 (13.5–14.1)	0.5 (0.4–0.5)	1.4 (1.3–1.5)	2.4 (2.3–2.5)	19.6 (19.2–20.0)
Suspected AF	2.2 (2.1–2.3)	1 [1–2]	8.2 (8.0–8.3)	0.4 (0.4–0.4)	8.5 (8.2–8.7)	0.2 (0.2–0.3)	0.8 (0.8–0.9)	1.2 (1.1–1.2)	19.3 (19.0–19.6)
Syncope	3.4 (3.3–3.5)	1 [1–4]	5.8 (5.7–5.8)	0.3 (0.3–0.3)	13.3 (13.1–13.4)	2.3 (2.2–2.3)	0.7 (0.7–0.8)	3.7 (3.6–3.8)	26.0 (25.8–26.2)
VT	2.1 (1.9–2.2)	1 [1–1]	5.0 (4.8–5.2)	0.2 (0.2–0.2)	52.9 (52.2–53.6)	0.2 (0.2–0.3)	0.9 (0.8–1.0)	4.7 (4.5–4.9)	64.0 (63.2–64.7)

AF = atrial fibrillation; AT = atrial tachycardia; brady = bradycardia; CI = confidence interval; IQR = interquartile range; VT = ventricular tachycardia.

### Staff time for ICM management

The overall volume of received transmissions required approximately 407 minutes per patient-year to be reviewed (3.0 transmissions per patient-month × 11.3 minutes × 12 months). In addition, device reprogramming occurred at a rate of 0.8 per patient-year, of which 61% were performed in-office (given that only 39% were conducted remotely). This corresponded to an additional 20 minutes per patient-year of staff time (0.8 × 61% × 39.9 minutes). This yielded an estimated total annual staff time of 7.1 hours for a patient with an ICM (0.36 full-time equivalent per 100 patients). In the group of patients who underwent reprogramming (38% at 1 year), the median reduction of transmissions was 57%, resulting in a potential annual saving of 1.5 hours of staff time per patient (38% × 3.0 transmissions per patient-month × 57% × 11.3 minutes × 12 months), that is, approximately 21% of the total staff time. The adoption of an alert-based review strategy with the elimination of scheduled transmissions would result in 2.1 hours per patient saved per year (3.0 transmissions per patient-month × 31% × 11.3 minutes × 12 months). Moreover, the full adoption of remote reprogramming would allow for saving the time spent on in-office visits. Thus, the total annual saving would be 2.4 hours of staff time per patient (2.1 hours + 20 minutes per patient-year), that is, approximately 34% of the total staff time. In our cohort and for the duration of observation, eliminating scheduled transmissions would have saved an estimated 1098 staff hours (5832 transmissions × 11.3 minutes). In addition, full adoption of remote reprogramming in place of in-office visits would have saved a further 117 hours (245 in-office reprogramming events: 245 × 39.9 minutes vs 245 × 11.3 minutes). Overall, these measures could have reduced staff workload by approximately 1215 hours.

## Discussion

In our study, we found that ICM reprogramming occurred in approximately 17% of patients within the first month after implantation and in 38% within 1 year. The timing of the first reprogramming varied by indication, with patients implanted for uncodified reasons experiencing earlier adjustments. Reprogramming was most often performed to lower detection sensitivity, thereby increasing the stringency of alert criteria. Remote reprogramming was used in only 39% of cases. The overall transmission rate was 3.0 per patient-month, with higher rates observed in patients implanted for palpitations and syncope. Although most transmissions were triggered by alerts, scheduled transmissions accounted for 31%. Among patients who underwent reprogramming, we observed a median reduction in transmissions of approximately 57%. In this context, these findings build on our preliminary study of the first enrolled cohort,^6^ in which LUX-Dx implantation was shown to be efficient and straightforward, yielding favorable postimplantation sensing values (R-wave amplitude and P-wave visibility) and receiving positive feedback from both operators and patients.

Previous studies have highlighted that ICMs generate a high volume of transmissions, necessitating remote episode review and adjudication, which places a substantial burden on follow-up resources in clinical centers.^1^^,^^2^^,^^7^ To address this challenge, manufacturers have enhanced device accuracy by developing and implementing sophisticated detection algorithms, yielding positive results. Notably, recent advancements in artificial intelligence have led to the development of dual-stage algorithms that significantly reduce false-positive alerts. These systems, such as those implemented in the LUX-Dx and LINQ II devices (Medtronic), use neural networks trained on extensive datasets to distinguish between clinically relevant events and benign occurrences, thereby decreasing alert burden without compromising the detection of true arrhythmic events.^4^ However, transmission volume is influenced not only by false-positive detections but also by repeated alerts for previously diagnosed, nonactionable events.

Current guidelines recommend reprogramming in cases of frequent false-positive or nonactionable alerts. Strategic reprogramming has been shown to effectively reduce transmission volumes,^8^ although it may require additional office visits. To mitigate this burden, modern ICMs have incorporated remote programming capabilities, allowing for alert optimization without in-person consultations. This aligns with recommendations suggesting that routine in-office visits are unnecessary for the ongoing management of ICM patients.^2^

We observed that the need for reprogramming was more pronounced in cases where ICMs recorded high volumes of detected episodes. This likely reflects efforts to manage inappropriate or nonactionable activations, as well as adjustments in monitoring needs, such as tracking arrhythmia progression after an initial AF diagnosis. In our study, reprogramming was most commonly aimed at reducing detection sensitivity or disabling detection altogether. Given that initial settings typically align with the nominal ICM configuration, this finding underscores the role of reprogramming in enhancing efficiency and reducing transmission burden.^4^^,^^8^

Despite its potential benefits, remote reprogramming was used in only 39% of cases. The overall reprogramming rate during follow-up was comparable with previous studies, with Seiler^9^ and Mahajan^3^ reporting a rate of 24% at 6 months. However, remote reprogramming was more widely used in their cohorts, at 75% and 82%, respectively. The preference for in-office vs remote reprogramming likely depends on the patient population, timing of reprogramming, and length of follow-up. In-office adjustments may be favored for symptomatic patients who present to the hospital independently or for actionable alerts requiring direct clinical assessment. In addition, the adoption of remote monitoring practices remains more limited in Europe owing to various logistical, economical, and regulatory factors.^10^^,^^11^

Overall, the most frequent reprogramming involved reducing sensitivity for bradyarrhythmia detection, particularly in patients with syncope or an “other” indication for implantation. This contrasts with previous studies in which AF detection parameters were most frequently adjusted.^4^^,^^9^ However, those studies included a higher proportion of patients implanted for suspected AF, which may explain the difference in reprogramming trends. Other parameters related to signal sensing were modified less frequently. One exception was sensing amplitude, which was sometimes lowered to increase sensitivity and prevent undersensing in patients implanted for VT monitoring; however, this adjustment was observed in fewer than 10% of cases.

The volume of alert transmissions is determined by the number of recorded episodes, which depends on the programmed detection sensitivity, as well as the activation status of each alert. We observed a high number of patient-triggered transmissions in the first days of follow-up, likely owing to patients’ initial need to familiarize themselves with the system. Although better patient training could help reduce these early transmissions, they do not seem to persist over time and therefore do not significantly contribute to the overall monthly transmission volume.

Regarding other alerts, our analysis of the time to the first event showed that the proportion of patients experiencing alerts within 1 year did not exceed 26% (eg, for VT). The discrepancy between this figure—representing the number of patients with a first recording for a potential initial diagnosis—and the total volume of transmissions received by the remote clinic highlights the potential workload reduction achievable by reprogramming the device to limit the transmission of known and nonactionable events.

The rate of received transmissions was approximately 3 per patient-month, consistent with previous studies (findings from published ICM studies on the volume of received transmissions, alerts, and recorded episodes are presented in Supplemental Table 2).^4^^,^^7^^,^^9^^,^^12^, ^13^, ^14^, ^15^, ^16^, ^17^, ^18^, ^19^, ^20^, ^21^ As expected, transmission volume varied based on the indication for ICM implantation. The LUX-Dx ICM automatically customizes detection parameters according to the primary monitoring indication set at enrollment, aligning with recommendations to tailor programming^2^ without requiring manual deviation from nominal settings. Nonetheless, it is reasonable to assume that more extensive customization of device settings at the time of implantation could reduce the need for early reprogramming during follow-up and immediately lower the volume of transmissions received. In our study, bradyarrhythmia episodes were frequently detected, with ICMs typically programmed to identify bradycardias of <40 beats per minute lasting longer than 1 second. This setting was often adjusted during reprogramming to reduce sensitivity, thereby decreasing unnecessary ICM activations.

The effectiveness of reprogramming was further confirmed by our paired pre- vs postreprogramming analysis, which demonstrated a significant reduction in recorded episodes, alert generation, and the overall number of transmissions requiring review.

Previous studies have shown that the LUX-Dx system supports high levels of remote monitoring, minimizing transmission failures and maintaining continuous connectivity throughout the monitoring period.^5^ This addresses transmission delays reported with earlier systems^22^^,^^23^ and plays a role in potentially reducing transmission volume. Notably, guidelines allow for the elimination of scheduled transmissions in cases of uninterrupted connectivity.^2^ However, our findings indicate that scheduled transmissions remain frequently programmed at 30-day intervals. Eliminating these transmissions could reduce the overall volume of transmissions by up to 31%.

The estimated resource requirement for remote management of ICM patients at the study centers was approximately 7.1 hours per patient-year, accounting for both remote transmission review and in-office reprogramming. This figure is comparable with Seiler’s^9^ reported estimate of 8.6 hours per patient-year.

The reduction in transmissions after reprogramming translated into a 21% decrease in overall staff workload. It is reasonable to assume that a broader implementation of reprogramming aimed at eliminating nonactionable alerts could further alleviate the burden of patient management, consistent with previous studies.^12^ However, it should be acknowledged that overly aggressive reprogramming may carry the risk of attenuating device sensitivity and potentially delaying or missing the detection of clinically relevant events. This highlights the importance of adhering to guideline recommendations,^2^ which emphasize individualized, patient-tailored programming to balance workload reduction with the need to preserve diagnostic accuracy. Moreover, the most immediate and impactful strategy for improving efficiency would be adopting a fully alert-based approach, eliminating scheduled transmissions, and maximizing the use of remote reprogramming. Together, these measures could potentially reduce the workload associated with ICM follow-up by one-third.

### Limitations

Our study has several potential limitations. This was a retrospective analysis of clinical data collected prospectively in real-world practice. Apart from the initial indication for monitoring, we did not capture the specific rationale behind physicians’ programming decisions. In addition, our analysis was limited to a median follow-up duration of 9 months, and both the utilization of remote programming and the frequency of ICM-detected events may differ over a longer observation period.

Furthermore, device reprogramming during follow-up was performed at the discretion of the operator, potentially leading to an underutilization of customized programming. As a result, our study may underestimate the full impact of the remote reprogramming feature in reducing ICM transmission burden. Finally, this study did not include a centralized adjudication of device-recorded events; therefore, we were unable to assess the positive predictive value of alerts or quantify the rate of false-positive or false-negative transmissions by event category.

## Conclusion

Our study highlights the significant role of ICM reprogramming in optimizing device performance and reducing the burden of remote monitoring. The remote reprogramming feature—currently used in 39% of cases—holds potential for broader adoption to optimize device management by reducing the need for in-office visits. Our findings also emphasize the potential for further efficiency gains by shifting to a fully alert-based monitoring strategy and eliminating scheduled transmissions, which could reduce follow-up workload by up to one-third.

## Disclosures

M.C. and S.V. are employees of Boston Scientific. G.C. has received consultancy fees from Bristol Myers Squibb and research grants from Boston Scientific Inc and Johnson & Johnson. The other authors report no conflicts.

## Figures and Tables

**Supplementary Figure 1:**