# Supplementary material for: Real-world use of insertable cardiac monitor remote programming: A multicenter European experience
Source: Heart Rhythm O2. 2025 Sep 1;6(11):1735–42. doi: 10.1016/j.hroo.2025.08.035 (PMC12675123; doi:10.1016/j.hroo.2025.08.035)
Supplement: Appendix [file mmc1.docx]

**APPENDIX:**

List of participating centers

- Hôpital de la Croix Rousse et Hôpital Lyon Sud, Hospices Civils de Lyon, Lyon, France: Mathieu Montoy, Samir Fareh, Paul Charles
- Istituto Cardiocentro Ticino, Lugano, Svizzera: Maria Luce Caputo, Marco Bergonti, Tardu Özkartal, Giulio Conte
- “Pineta Grande” Hospital, Castelvolturno (CE), Italy: Stefano Nardi, Luigi Argenziano, Vittoria Marino
- Clinica Sanatrix, Naples, Italy: Luigi Argenziano, Stefano Nardi, Vittoria Marino
- Ospedale Maggiore di Lodi, Lodi, Italy: Francesco Villella, Luca Poggio
- Ospedale “Don Calabria” Sacro Cuore, Negra (VR): Giulio Molon, Alessandro Costa
- Fatebenefratelli Hospital, Naples, Italy: Giuseppe Franzone, Cristina De Colle, Vittoria Miano, Fernando Scala
- Casa di Cura “Villa Azzurra”, Siracusa, Italy: Aldo Centaro, Nicole Monaca, Antonino Frasca, Alessandro Diamante
- “Santissima Annunziata” Hospital, Taranto, Italy: Mandurino Cosimo, Giovanna Rodio, Michele Scolletta, Leonardo Di Gregorio, Marilisa Ludovico, Giovanni Luzzi
- Department of Cardiovascular, Respiratory, Nephrological, Anesthesiological and Geriatric Sciences, "Sapienza" University of Rome, Policlinico Umberto I, Rome, Italy: Agostino Piro, Marco Valerio Mariani, Nicola Pierucci, Carlo Lavalle
- Arrhythmia and Electrophysiology Unit, Careggi University Hospital, Florence, Italy: Paolo Pieragnoli, Giuseppe Ricciardi, Laura Perrotta, Gabriele Bambagioni, Luca Checchi
- Ospedale Isola Tiberina - Gemelli Isola, Rome, Italy: Marco Polselli, Antonio Bisignani, Michele Magnocavallo
- “Bambin Gesù” Pediatric Hospital, Rome, Italy: Cristina Raimondo, Ilaria Tamburri, Fabio Anselmo Saputo, Marta Campisi, Massimo Stefano Silvetti
- San Pietro-Fatebenefratelli Hospital, Rome, Italy: Barbara Romani, Jacopo Costantino, Daniele Porcelli
- “L. Bonomo” Hospital, Andria, Italy: Gianluca Antonio Robles, Pierpaolo Vitti, Francesco Musaico, Domenico Gianfrancesco
- Ospedale S. Jacopo, Pistoia, Italy: Andrea Boncompagni
- Ospedale Carlo Poma, Mantova, Italy: Mattia Strazzanti, Caterina Gola, Anna Branzanti
- P.O. Di Venere, Bari, Italy: Massimo Vincenzo Bonfantino, Rosanna Valecce
- Ospedale S. Maria Goretti, Latina, Italy: Rita Di Rosa, Emanuela Viazzo, Mario Ferraiolo
- Azienda Ospedaliero Universitaria di Siena, Italy: Claudia Baiocchi, Stefano Lunghetti, Amato Santoro.
- Ospedale A. Perrino, Brindisi, Italy: Giovanni Caroli
- Azienda Ospedaliero Universitaria di Bari, Italy: Vincenzo Ezio Santobuono, Riccardo Memeo
- Ospedale “G.B. Grassi”, Rome, Italy: Karim Mahfouz, Stefania Gentile, Luca Sangiovanni, Luca Santini
