# Supplementary material for: Real-world use of insertable cardiac monitor remote programming: A multicenter European experience
Source: Heart Rhythm O2. 2025 Sep 1;6(11):1735–42. doi: 10.1016/j.hroo.2025.08.035 (PMC12675123; doi:10.1016/j.hroo.2025.08.035)
Supplement: Supplementary Figure 1 [file mmc2.docx]

**Supplemental Figure 1.** Percentage of patients with parameter settings changed from implantation programming to the last transmission. Patients are stratified according to the indications for ICM implantation.
