# Supplementary material for: Real-world use of insertable cardiac monitor remote programming: A multicenter European experience
Source: Heart Rhythm O2. 2025 Sep 1;6(11):1735–42. doi: 10.1016/j.hroo.2025.08.035 (PMC12675123; doi:10.1016/j.hroo.2025.08.035)
Supplement: Supplementary Table 1 [file mmc3.docx]

**Supplemental Table 1.** Programming of ICM settings at implantation.

| **Parameter** | | **Settings** | | | | | | |
| --- | --- | --- | --- | --- | --- | --- | --- | --- |
| **Sensing** | Blank After Sense | **160 ms:** 697 |  |  |  |  |  |  |
|  | Sensitivity | **0.037 mV:** 697 |  |  |  |  |  |  |
|  | Morphology Assessment | **OFF:** 0 | **ON:** 697 |  |  |  |  |  |
| **Brady** | Detection | **OFF:** 0 | **ON:** 697 |  |  |  |  |  |
|  | Rate | **30 bpm:** 8 | **40 bpm:** 689 |  |  |  |  |  |
|  | Duration | **1 s:** 690 | **2 s:** 1 | **3 s:** 2 | **5 s:** 1 | **10 s:** 2 | **30 s:** 1 |  |
|  | Alert status | **OFF:** 254 | **ON-Red:** 325 | **ON-Yellow:** 118 |  |  |  |  |
| **Tachy** | Detection | **OFF:** 0 | **ON:** 697 |  |  |  |  |  |
|  | Rate | **140 bpm:** 1 | **150 bpm:** 1 | **160 bpm:** 1 | **165 bpm:** 1 | **170 bpm:** 691 | **180 bpm:** 1 | **200 bpm:** 1 |
|  | Duration | **5 s:** 693 | **10 s:** 2 | **15 s:** 1 | **20 s:** 1 |  |  |  |
|  | Response | **LESS:** 202 | **BALANCED:** 113 | **MORE:** 382 |  |  |  |  |
|  | Alert status | **OFF:** 61 | **ON-Red:** 614 | **ON-Yellow:** 22 |  |  |  |  |
| **Pause** | Detection | **OFF:** 0 | **ON:** 697 |  |  |  |  |  |
|  | Duration | **3 s:** 695 | **4.5 s:** 2 |  |  |  |  |  |
|  | Response | **LESS:** 251 | **BALANCED:** 113 | **MORE:** 333 |  |  |  |  |
|  | Alert status | **OFF:** 93 | **ON-Red:** 350 | **ON-Yellow:** 254 |  |  |  |  |
| **Symptom** | Recording | **OFF:** 0 | **ON:** 697 |  |  |  |  |  |
|  | Max Recordings/day | **4 (7.5 min):** 696 | **6 (5 min):** 1 |  |  |  |  |  |
|  | Alert status | Symptoms | | |  | Symptoms + Event detected | | |
|  |  | **OFF:** 280 | **ON-Red:** 2 | **ON-Yellow:** 415 |  | **OFF:** 33 | **ON-Red:** 552 | **ON-Yellow:** 112 |
| **AT** | Detection | **OFF:** 4 | **ON:** 693 |  |  |  |  |  |
|  | Rate | **110 bpm:** 695 | **140 bpm:** 1 | **160 bpm:** 1 |  |  |  |  |
|  | Duration | **240 min:** 697 |  |  |  |  |  |  |
|  | Alert status | **OFF:** 678 | **ON-Red:** 0 | **ON-Yellow:** 19 |  |  |  |  |
| **AF** | Detection | **OFF:** 4 | **ON:** 693 |  |  |  |  |  |
|  | Response | **LEAST:** 0 | **LESS:** 381 | **BALANCED:** 146 | **MORE:** 170 | **MOST:** 0 |  |  |
|  | Duration | **4 min:** 170 | **6 min:** 51 | **10 min:** 476 |  |  |  |  |
|  | Alert status (Events) | **OFF:** 490 | **ON-Red:** 155 | **ON-Yellow:** 52 |  |  |  |  |
|  | Alert status (Burden) | **OFF:** 654 | **ON-Red:** 3 | **ON-Yellow:** 40 | **ON (1 hour):** 39 | **ON (6 hours):** 3 | **ON (12 hours):** 1 |  |
| **Scheduled transmissions** | | **Every 7 days:** 51 | **Every 15 days:** 1 | **Every month:** 644 | **Every 6 months:** 1 | |  |  |

**Supplemental Table 2.** Findings from published ICM studies on the volume of received transmissions, alerts and recorded episodes.

| Author [year] | Device | Sample Size | Indication for ICM | Follow-up Duration | Transmissions | Alerts | Recorded Episodes |
| --- | --- | --- | --- | --- | --- | --- | --- |
| Søgaard et al. [2019] (15) | BioMonitor II | 212 | Post MI | Average 13 months | 80,404 messages | 22,158 messages with sECGs | 78,541 sECGs |
| Ip et al. [2020] (16) | Confirm Rx, LINQ | 142 | - Stroke 69% - Syncope 24% - Palpitations 7% | 7.1±3.5 months |  | 3510 events  (25.5±45.6 vs 0.9±1.1 per patient-month) |  |
| Muntané-Carol et al. [2021] (17) | Reveal XT or LINQ | 104 | New-onset persistent LBBB post-TAVR | 12 months |  | 1649 arrhythmic events  5 (IQR: 1–13) events per patient |  |
| Jansson et al. [2021] (18) | Reveal XT | 150 | Paroxysmal or persistent AF | 2.0±0.5 months |  | Median number of AF episodes per month 3.6 (IQR: 0.98–16.40) |  |
| Reinsch et al. [2022] (19) | BioMonitor III | 30 | - Syncope 80% - Cryptogenic stroke 16% - AF monitoring 4% | 99 days (IQR: 92–107) | 10225 true episodes and misclassifications |  | Median number of sECGs per patient: 6 (IQR: 1.5–52.5). |
| O'Shea et al. [2021] (7) | ICMs managed with PaceMate | 5,032 |  | 1 year |  | 41,454 alerts  Alerts per year 4.0 (IQR: 2–12) |  |
| Afzal [2021] (14) | LINQ, ConfirmRx, BioMonitor II/III | 1,811 | - AF surveillance 42% - Cryptogenic stroke 31% - Syncope 15% - Palpitations 9% - Miscellaneous 2% | 4 weeks | 1,457 (995 scheduled) | 462 | Total arrhythmia episodes with complete ECGs 9,222 |
| Quartieri et al. [2022] (20) | Confirm Rx | 20 | - Syncope 75% - Risk for arrhythmias 15% - Atrial fibrillation 5% - Cryptogenic stroke 5% | 23 months |  |  | 2261 sECGs with arrhythmia detections or patient symptoms |
| Guarracini et al. [2023] (21) | BioMonitor III/IIIm | 119 | - Syncope 38% - AF management 31% - Cryptogenic stroke 23% - Pre-syncope 8% | 371 |  | Median number of arrhythmic events per patient-year 14.2 (IQR: 1.8–126) | 14,136 episodes |
| Gala et al. [2024] (22) | Confirm Rx | 232 | - Syncope 65% - Palpitations 16% - Other 10% - Suspected AF 7% - AF management 3% | 18 (IQR: 10–22) |  |  | 16,230 AF episodes  Median number per patient 10 (3–58) |
| Bisignani et al. [2024] (23) | BioMonitor IIIm | 368 | - Syncope or Pre-syncope 42% - Cryptogenic stroke 31% | Total Observation Period 419 years | 143,096  Number per patient-year 341.9 |  | 61,517 sECGs,  Number per patient-year 147 |
| Seiler et al. [2024] (9) | LINQ II and LINQ TR | 19,525 and  117,964 | - AF surveillance >40% - AF management 25% - Syncope 23% |  |  | Alerts per patient-month 3.32±6.82 |  |
| Neiman et al. [2024] (4) | LINQ II and LUX-Dx | 117 and 105 | - Syncope 34% - Cryptogenic stroke 27% - AF management 15% | 98 days (IQR: 71–100) and  96 days (IQR: 89–96) | 331 and 334 | 239 and 300  Alert transmissions per day: 0.13 (IQR: 0.09–0.24) and 0.15 (IQR: 0.05–0.36) |  |
